# Factors associated with willingness and preferences to attend family services in Hong Kong: A population-based survey

**DOI:** 10.3389/fpubh.2023.1057164

**Published:** 2023-02-09

**Authors:** Yingpei Zeng, Weijie Gong, Agnes Yuen Kwan Lai, Shirley Man Man Sit, Man Ping Wang, Sai Yin Ho, Tai Hing Lam

**Affiliations:** ^1^School of Nursing, The University of Hong Kong, Hong Kong, Hong Kong SAR, China; ^2^Department of General Practice, Health Science Center, Shenzhen University, Shenzhen, China; ^3^School of Public Health, The University of Hong Kong, Hong Kong, Hong Kong SAR, China

**Keywords:** family wellbeing, family communication, family support, social service, family service

## Abstract

**Objective:**

Family services are open to the community at large as well as vulnerable groups; however, little is known about the willingness of communities to attend such services. We investigated the willingness and preferences to attend family services and their associated factors (including sociodemographic characteristics, family wellbeing, and family communication quality) in Hong Kong.

**Methods:**

A population-based survey was conducted on residents aged over 18 years from February to March 2021. Data included sociodemographic characteristics (sex, age, education, housing type, monthly household income, and the number of cohabitants), willingness to attend family services to promote family relationships (yes/no), family service preferences (healthy living, emotion management, family communication promotion, stress management, parent-child activities, family relationship fostering, family life education, and social network building; each yes/no), family wellbeing, and family communication quality (both scores 0–10). Family wellbeing was assessed using the average scores of perceived family harmony, happiness and health (each score 0–10). Higher scores indicate better family wellbeing or family communication quality. Prevalence estimates were weighted by sex, age and educational level of the general population. Adjusted prevalence ratios (aPR) for the willingness and preferences to attend family services were calculated in relation to sociodemographic characteristics, family wellbeing, and family communication quality.

**Results:**

Overall, 22.1% (1,355/6,134) and 51.6% (996/1,930) of respondents were willing to attend family services to promote relationships or when facing problems, respectively. Older age (aPR = 1.37–2.30, *P* < 0.001–0.034) and having four or more cohabitants (aPR = 1.44–1.53, *P* = 0.002–0.003) were associated with increased aPR of willingness for both situations. Lower family wellbeing and communication quality were associated with lower aPR for such willingness (aPR = 0.43–0.86, *P* = 0.018–<0.001). Lower family wellbeing and communication quality were associated with preferences for emotion and stress management, family communication promotion, and social network building (aPR = 1.23–1.63, *P* = 0.017–<0.001).

**Conclusions:**

Lower levels of family wellbeing and communication quality were associated with unwillingness to attend family services and preferences for emotion and stress management, family communication promotion, and social network building.

## Introduction

Family wellbeing is the foundation of a harmonious society that promotes psychological health and individual flourishing (1–3). Family communication, whether verbal or non-verbal, is key to maintaining family relationships through the sharing of meaning, thoughts, attitudes, and benefits (4, 5). Good family relationships come from the support of family members and external social support (1, 6) the latter providing mechanisms of coping with adversity (7).

Family services aims to strengthen the bonding, support, coping skills and wellbeing of families (8, 9). Family services generally include: family support services provided to the general population to enhance family roles, family-centered services provided to families at risk to strengthen stability when facing problems, and intensive family preservation services provided to families in crisis using comprehensive home-based, concrete, and therapeutic interventions (5–20 h per week for 4-8 weeks). We have searched Web of Science, PubMed and ScienceDirect using keywords of “family service,” “social service,” “family relationships,” and “wellbeing” up to 18 December 2022, and found no reports on the prevalence and associated factors (e.g., sociodemographic characteristics) of willingness and preferences to attend family services.

During the COVID-19 pandemic, families may have got separated because of social distancing restrictions (10). Family services provide on-site and online interventions to promote family wellbeing and communication quality, hence adversity coping capabilities (11). According to the inverse care law (12), medical care would be less utilized by socially disadvantaged people before and amidst the COVID-19 pandemic (13, 14). Few studies have examined the inverse care law in family services. We hypothesized that lower family wellbeing and communication quality are positively associated with unwillingness to attend family services (inverse family care law).

Hong Kong, the most westernized city in China, emphasizes collectivism and family cohesion (15). Family services are operated by 12 non-governmental organizations and the Social Welfare Department. These services, located in all 18 districts of Hong Kong, are accessible to families and individuals from different socioeconomic levels (16). Identifying the factors associated with family service use can help understand service needs and provide better services through personalized assistance (9). The sociodemographic factors associated with family service use in vulnerable groups are already well-defined (17, 18). We examined the associations of sociodemographic characteristics, family wellbeing, and family communication quality with the willingness to attend family services in the general population. Preferences of family services were also explored.

## Participants and methods

### Sampling methods

Under the Jockey Club SMART Family-Link project, the population-based Family amidst COVID-19 survey 2 (FamCov-2) was conducted from 22 February to 23 March 2021, when the fourth wave of the COVID-19 pandemic was under control. Eligible respondents were Hong Kong residents aged over 18 years who could read or communicate in Cantonese.

The Hong Kong Public Opinion Research Institute, a survey agency, was commissioned to conduct the survey online and *via* landline and mobile telephone numbers. Using known prefixes, landline numbers were randomly generated from telecommunication service providers under the Numbering Plan of the Office of the Communication Authority. Invalid numbers were excluded from the list. Mobile numbers were generated likewise. In the landline survey, the household members who would have their birthday next was selected. The survey included three subsets—family communication, COVID-19 information, and COVID-19 influences. Each subset comprised core questions answered by all respondents and random questions by one-third of the respondents. Details of these methods have been reported elsewhere (19, 20).

Of the 1,604 and 816 eligible respondents who answered the landline and mobile surveys, 1,022 (response rate: 63.7%) and 500 (61.3%) completed the entire survey, respectively, with a combined response rate of 62.9%. The survey agency sent email invitations to members of its probability and non-probability online panels with a link to the online survey. Of the 4,311 and 44,514 probability and non-probability panel members who opened the invitation emails, 641 (14.2%) and 5,372 (12.1%) respondents completed the entire survey, respectively. Totally 7,535 respondents were enrolled in the survey online and *via* landline and mobile telephone. All respondents provided informed consents before answering the survey. Ethics approval was granted by the Institutional Review Board of the University of Hong Kong/Hospital Authority Hong Kong West Cluster (IRB UW 20-651).

### Measurements

All respondents were asked “Would you like to participate in face-to-face or online activities (separate questions) organized by social welfare agencies to promote family relationships?” with responses of “Yes” or “No.” For those who answered “Yes,” we further asked the types of family services they preferred. The response options covered healthy living, emotion management, family communication promotion, stress management, parent-child activities, family relationship fostering, family life education, and social network building. The respondents could choose more than one option. In the subset of family communication, a random question asked if respondents were willing to attend face-to-face or online family services when facing family problems, with the response of “Yes” or “No.”

Family harmony, happiness, and health (3Hs) are the three core components of family wellbeing in Chinese culture (3, 21). Family wellbeing was assessed using the average scores of perceived family harmony, happiness, and health in our previous studies (15, 22), by asking “How healthy/happy/harmonious do you think your family is?” with a score ranging from 0 to 10. Family communication quality was assessed on a score of 0 to 10, as used in our previous study (23). A higher score indicates higher family wellbeing or family communication quality.

Data on sociodemographic characteristics were collected, including sex, age group (18–24, 25–44, 45–64, over 65 years), educational attainment (secondary or below, tertiary), housing type (rented, owned), monthly household income (HK$9,999 or less, HK$10,000–HK$39,999, HK$40,000 or above), the number of cohabitants (none, 1–3 people, four, or more people).

### Statistical analyses

The original data and prevalence estimates were weighted by sex, age, and educational attainment distribution of the 2019 Hong Kong census data (24). We created a composite variable for the willingness to attend family services to promote family relationships *via* face-to-face or online means, with responses of “Yes” (willing to attend services either *via* face-to-face or online means) or “No” (unwilling to attend by both means). A similar composite variable, willingness to attend family services when facing family problems, was also created. The scores for family wellbeing, and family communication quality were categorized into high (7–10), medium (4–6), and low (0–3). The sociodemographic characteristics, family wellbeing, and family communication quality of respondents were compared by their willingness to attend family services using chi-square tests and *t*-tests, as appropriate. Poisson regression with robust variance estimator (25) was used to yield adjusted prevalence ratios (aPR) for the willingness to attend and each preference of family services in relation to sociodemographic characteristics, family wellbeing, and family communication quality. All analyses were conducted using Stata version 15, with a 2-sided *P* < 0.05 indicating statistical significance.

## Results

Table 1 shows that, of the 6,134 respondents who answered the question of willingness to attend family services for promoting family relationships, 52.2% were female, 78.2% were aged 25–64 years, and 73.5% had tertiary education. The prevalence of the above characteristics was similar for the respondents (*n* = 1,930) in the subset of family communication, who answered about their willingness to attend family services when facing family problems. 22.1% (1,355/6,134) were willing to attend family services to promote family relationships and 51.6% (996/1,930) were willing to attend services when facing family problems. Such willingness was associated with age, having more cohabitants and with higher family wellbeing and family communication quality (*P* = 0.009– <0.001).

**Table 1:** Sociodemographic characteristics, family wellbeing, and family communication quality by willingness to attend family services *via* face-to-face or online means^a^.

**Variables, *n* (%)**	**Family services to promote family relationships**		**P^b^**	**Total (*****N*** = **6,134)**		**Family services when facing family problems**		**P^b^**	**Total (*****N*** = **1,930)**	
	**Yes (*****n*** = **1,355)**	**No (*****n*** = **4,779)**		**Unweighted**, ***n*** **(%)**	**Weighted**, ***n*** **(%)**^c^	**Yes (*****n*** = **996)**	**No (*****n*** = **934)**		**Unweighted**, ***n*** **(%)**	**Weighted**, ***n*** **(%)**^c^
Sex			0.002					0.711		
Male	698 (51.6)	2,232 (46.7)		2,930 (47.8)	2,930 (47.7)	484 (48.6)	446 (47.8)		930 (48.2)	930 (48.3)
Female	656 (48.4)	2,544 (53.3)		3,200 (52.2)	3,200 (52.3)	512 (51.4)	488 (52.2)		1,000 (51.8)	1,000 (51.7)
Age (years)			0.000					0.001		
18–24	60 (4.4)	442 (9.3)		502 (8.2)	502 (9.1)	51 (5.1)	89 (9.5)		140 (7.3)	140 (8.6)
25–44	586 (43.4)	1,846 (38.7)		2,432 (39.7)	2,432 (32.2)	420 (42.2)	363 (39.0)		783 (40.6)	783 (33.3)
45–64	526 (38.9)	1,831 (38.4)		2,357 (38.5)	2,357 (38.5)	397 (39.9)	345 (37.0)		742 (38.5)	742 (39.9)
≥65	180 (13.3)	651 (13.6)		831 (13.6)	831 (20.2)	127 (12.8)	135 (14.5)		262 (13.6)	262 (18.2)
Educational attainment			0.086					0.684		
Secondary or below	332 (24.7)	1,282 (27.0)		1,614 (26.5)	1,342 (61.5)	262 (26.5)	254 (27.4)		516 (27.0)	516 (67.2)
Tertiary	1,014 (75.3)	3,465 (73.0)		4,479 (73.5)	4,479 (35.6)	725 (73.5)	674 (72.6)		1,399 (73.0)	1,399 (32.8)
Housing type			0.422					0.560		
Rented	494 (36.7)	1,801 (37.9)		2,295 (37.6)	2,295 (40.1)	374 (37.7)	363 (39.0)		737 (38.4)	737 (43.2)
Owned	853 (63.3)	2,954 (62.1)		3,807 (62.4)	3,807 (59.9)	617 (62.3)	567 (61.0)		1,184 (61.6)	1,184 (56.8)
Monthly household income (HK$)^d^		0.144					0.032		
≤9,999	159 (13.1)	470 (11.5)		629 (11.9)	629 (14.7)	97 (11.1)	114 (14.4)		211 (12.7)	211 (16.8)
10,000–39,999	422 (34.8)	1,526 (37.4)		1,948 (36.8)	1,948 (48.7)	317 (36.2)	305 (38.6)		622 (37.3)	622 (49.1)
≥40,000	631 (52.1)	2,084 (51.1)		2,715 (51.3)	2,715 (36.6)	461 (52.7)	372 (47.0)		833 (50.0)	833 (34.1)
Number of cohabitants			0.001					0.001		
0	90 (6.6)	444 (9.3)		534 (8.7)	534 (8.1)	63 (6.3)	104 (11.1)		167 (8.6)	167 (7.1)
1–3	1,050 (77.5)	3,713 (77.7)		4,763 (77.6)	4,763 (76.1)	795 (79.8)	712 (76.2)		1,507 (78.1)	1,507 (76.8)
≥4	215 (15.9)	622 (13.0)		837 (13.7)	837 (15.8)	138 (13.9)	118 (12.7)		256 (13.3)	256 (16.1)
**Family wellbeing, mean (SD)**										
Overall	6.7 (1.8)	6.5 (2.0)	<0.001	6.5 (1.9)	6.5 (1.9)	6.8 (1.7)	6.2 (2.1)	<0.001	6.5 (1.9)	6.5 (1.9)
Family health	6.6 (1.9)	6.4 (2.1)	<0.001	6.4 (2.0)	6.4 (2.0)	6.7 (1.8)	6.1 (2.3)	<0.001	6.4 (2.1)	6.4 (2.0)
Family harmony	6.8 (1.9)	6.6 (2.1)	0.009	6.7 (2.1)	6.7 (2.1)	7.0 (1.8)	6.4 (2.3)	<0.001	6.7 (2.1)	6.7 (2.1)
Family happiness	6.7 (1.9)	6.4 (2.1)	<0.001	6.4 (2.1)	6.4 (2.1)	6.8 (1.8)	6.1 (2.3)	<0.001	6.5 (2.1)	6.4 (2.1)
Family communication quality, mean (SD)	6.2 (2.0)	6.0 (2.2)	0.001	6.1 (2.2)	6.1 (2.1)	6.4 (1.9)	5.7 (2.4)	<0.001	6.1 (2.2)	6.1 (2.1)

^a^Respondents with missing values were excluded.

^b^Chi-square tests or t-tests.

^c^Weighted by sex, age, and educational attainment distribution of Hong Kong census.

^d^HK $7.8 = US $1.

Table 2 shows that, after mutual adjustment, lower family wellbeing (aPR = 0.43–0.86, *P* = 0.018–<0.001) and family communication quality (aPR = 0.54–0.85, *P* = 0.001–<0.001) were negatively associated with the willingness to attend family services for promoting family relationships or when facing family problems. Older age (aPR = 1.37–2.30, *P* < 0.001–0.034) and having four or more cohabitants (aPR = 1.44–1.53, *P* = 0.002–0.003) were associated with increased aPR of willingness for both situations. For promoting family relationships, males (aPR = 1.13, 95% CI: 1.01, 1.24) and tertiary education (aPR = 1.14, 95% CI: 1.00, 1.30) were associated with the willingness to attend family services, whereas higher household income was associated with lower aPR of willingness (aPR = 0.78, 95% CI: 0.66, 0.93). We combined face-to-face and online means of family services as the results were similar (Supplementary Table 1).

**Table 2:** The associations of willingness to attend family services with sociodemographic characteristics, family wellbeing, and family communication quality, PR (95% CI).

	**Services to promote family relationship 1,355/6,134 (22.1%)**				**Services when facing with family problems 996/1,930 (51.6%)**			
	**Crude model**	***P***	**Adjusted model**	***P***	**Crude model**	***P***	**Adjusted model**	***P***
**Sex**								
Female	1		1		1		1	
Male	1.16 (1.06–1.28)	0.002	1.13 (1.01–1.24)	0.020	1.02 (0.93–1.11)	0.711	1.01 (0.92–1.11)	0.804
**Age (years)**								
18–24	1		1		1		1	
25–44	2.02 (1.57–2.58)	<0.001	2.30 (1.74–3.06)	<0.001	1.47 (1.17–1.85)	0.001	1.53 (1.18–1.98)	0.001
45–64	1.87 (1.46–2.40)	<0.001	2.15 (1.61–2.86)	<0.001	1.47 (1.17–1.85)	0.001	1.49 (1.15–1.94)	0.003
≥65	1.83 (1.38–2.37)	<0.001	1.99 (1.45–2.74)	<0.001	1.33 (1.03–1.71)	0.026	1.37 (1.02–1.84)	0.034
**Educational attainment**								
Secondary or below	1		1		1		1	
Tertiary	1.10 (0.99–1.23)	0.088	1.14 (1.00–1.30)	0.049	1.02 (0.92–1.13)	0.686	0.98 (0.87–1.10)	0.698
**Housing type**								
Rented	1		1		1		1	
Owned	1.04 (0.94–1.15)	0.422	1.03 (0.93–1.14)	0.591	1.03 (0.94–1.12)	0.562	1.00 (0.91–1.10)	0.960
**Monthly household income (HK$)** ^a^								
≤9,999	1		1		1		1	
10,000–39,999	0.86 (0.73–1.00)	0.057	0.78 (0.66–0.93)	0.004	1.11 (0.94–1.31)	0.222	1.02 (0.86–1.21)	0.807
≥40,000	0.92 (0.79–1.07)	0.275	0.78 (0.66–0.92)	0.003	1.20 (1.03–1.41)	0.022	1.08 (0.91–1.28)	0.402
**Number of cohabitants**								
0	1		1		1		1	
1–3	1.31 (1.08–1.59)	0.007	1.19 (0.96–1.47)	0.115	1.40 (1.14–1.71)	0.001	1.50 (1.16–1.94)	0.002
≥4	1.52 (1.22–1.90)	<0.001	1.44 (1.13–1.83)	0.003	1.43 (1.14–1.79)	0.002	1.53 (1.16–2.02)	0.003
**Family wellbeing** ^b, c^								
High	1		1		1		1	
Medium	0.87 (0.78–0.97)	0.013	0.86 (0.77–0.98)	0.018	0.80 (0.72–0.89)	<0.001	0.82 (0.74–0.92)	0.001
Low	0.71 (0.57–0.89)	0.003	0.69 (0.54–0.88)	0.003	0.44 (0.32–0.60)	<0.001	0.43 (0.31–0.60)	<0.001
**Family communication quality** ^b, c^								
High	1		1		1		1	
Medium	0.92 (0.83–1.01)	0.084	0.92 (0.83–1.02)	0.128	0.85 (0.77–0.93)	<0.001	0.85 (0.77–0.94)	0.001
Low	0.72 (0.61–0.85)	<0.001	0.72 (0.60–0.86)	<0.001	0.56 (0.47–0.67)	<0.001	0.54 (0.45–0.66)	<0.001

PR, Prevalence ratio; CI, Confidence interval. All sociodemographic variables were mutually adjusted in multivariate Poisson models.

^a^HK $7.8 = US $1.

^b^Adjusting for sex, age, educational attainment, housing type, monthly household income, and number of cohabitants.

^c^Scale: 0–10, high (7–10), medium (4–6), low (0–3).

Table 3 shows that, of 1,355 respondents who were willing to attend family services to promote family relationships, healthy living (56.8%) was the most popular preference, followed by emotion management (47.2%) and family communication promotion (44.1%). Respondents with lower family wellbeing and family communication quality preferred family services on emotion management, stress management, family communication promotion, and social network building (aPR = 1.23–1.63, *P* = 0.017–<0.001), but had less interests in parent-child activities (aPR = 0.56–0.84, *P* = 0.017–<0.001). Males preferred the topic of social network building (aPR = 1.26, 95% CI: 1.04, 1.53). Respondents aged over 65 years had a higher preference for healthy living (aPR = 1.59, 95% CI: 1.15, 2.21) and a lower preference for stress management (aPR = 0.49, 95% CI: 0.33, 0.74). Higher preference for parent-child activities was shown in adults aged 25–44 years (aPR = 1.79, 95% CI: 1.12, 2.86), whereas lower preference was reported by those with tertiary education (aPR = 0.66, 95% CI: 0.55, 0.79). Those who had four or more cohabitants showed a higher preference for parent-child activities (aPR = 2.47, 95% CI: 1.47, 4.15) and a lower preference for social network building (aPR = 0.57, 95% CI: 0.37, 0.89).

**Table 3:** The associations of preferences of family services with sociodemographic characteristics, family wellbeing, and family communication quality, aPR (95% CI) (*N* = 1,355).

	**Healthy living**	**Emotion management**	**Family communication promotion**	**Stress management**	**Parent-child activities**	**Family relationship fostering**	**Family life education**	**Social network building**
*n*/%	769 (56.8)	639 (47.2)	597 (44.1)	563 (41.6)	517 (38.2)	528 (39.0)	422 (31.1)	359 (26.5)
**Sex**								
Female	1							
Male	0.93 (0.84–1.02)	1.05 (0.93–1.19)	1.00 (0.88–1.13)	0.97 (0.85–1.12)	0.92 (0.80–1.05)	1.14 (0.99–1.32)	1.10 (0.93–1.31)	1.26 (1.04–1.53)^*^
**Age (years)**								
18–24	1							
25–44	0.97 (0.71–1.33)	1.17 (0.83–1.65)	0.87 (0.65–1.19)	0.87 (0.64–1.19)	1.79 (1.12–2.86)^*^	1.14 (0.77–1.68)	1.09 (0.69–1.71)	1.03 (0.59–1.80)
45–64	1.32 (0.96–1.80)	1.14 (0.80–1.61)	0.87 (0.64–1.19)	0.87 (0.64–1.19)	1.08 (0.66–1.75)	0.93 (0.62–1.38)	0.97 (0.61–1.54)	1.05 (0.60–1.83)
≥65	1.59 (1.15–2.21)^**^	0.67 (0.44–1.02)	0.87 (0.61–1.25)	0.49 (0.33–0.74)^**^	0.60 (0.33–1.08)	1.00 (0.64–1.57)	0.74 (0.43–1.29)	1.40 (0.77–2.54)
**Educational attainment**								
Secondary or below	1							
Tertiary	1.03 (0.91–1.16)	0.96 (0.82–1.12)	0.96 (0.80–1.14)	0.97 (0.81–1.16)	0.66 (0.55–0.79)^***^	0.94 (0.77–1.14)	0.87 (0.69–1.09)	0.95 (0.74–1.21)
**Housing type**								
Rented	1							
Owned	1.00 (0.90–1.11)	1.01 (0.89–1.14)	0.98 (0.86–1.12)	0.98 (0.85–1.13)	0.91 (0.79–1.05)	1.05 (0.90–1.22)	1.10 (0.92–1.32)	1.07 (0.88–1.31)
**Monthly household income (HK$)** ^a^								
≤9,999	1							
10,000–39,999	1.02 (0.88–1.19)	0.99 (0.80–1.22)	1.20 (0.93–1.53)	0.90 (0.72–1.14)	0.95 (0.70–1.29)	1.10 (0.84–1.44)	0.98 (0.71–1.36)	1.04 (0.77–1.40)
≥40,000	0.99 (0.85–1.16)	0.86 (0.70–1.07)	1.27 (0.99–1.64)	0.84 (0.66–1.06)	1.34 (0.99–1.82)	1.15 (0.88–1.51)	1.11 (0.80–1.53)	0.75 (0.54–1.05)
**Number of cohabitants**								
0	1							
1–3	1.04 (0.86–1.25)	0.83 (0.67–1.04)	1.21 (0.87–1.69)	0.91 (0.69–1.19)	1.63 (0.98–2.72)	0.90 (0.66–1.22)	1.19 (0.77–1.84)	0.76 (0.54–1.07)
≥4	0.94 (0.75–1.18)	0.82 (0.63–1.07)	1.24 (0.87–1.78)	0.88 (0.65–1.20)	2.47 (1.47–4.15)^**^	1.00 (0.71–1.42)	1.35 (0.85–2.16)	0.57 (0.37–0.89)^*^
**Family wellbeing** ^b, c^								
High	1							
Medium	0.93 (0.83–1.06)	1.32 (1.15–1.51)^***^	1.33(1.16–1.53)^***^	1.33 (1.14–1.55)^***^	0.67 (0.56–0.80)^***^	1.14 (0.97–1.34)	0.92 (0.75–1.13)	1.18 (0.94–1.48)
Low	0.94 (0.73–1.21)	1.54 (1.25–1.90)^***^	1.21 (0.89–1.63)	1.38 (1.06–1.79)^*^	0.73 (0.49–1.09)	0.90 (0.62–1.31)	1.10 (0.75–1.60)	1.63 (1.13–2.34)^**^
**Family communication quality** ^b, c^								
High	1							
Medium	1.00 (0.90–1.11)	1.26 (1.11–1.44)^***^	1.34(1.17–1.53)^***^	1.23 (1.06–1.42)^**^	0.84 (0.72–0.97)^*^	1.14 (0.98–1.33)	1.07 (0.89–1.28)	1.21 (0.98–1.49)
Low	0.93 (0.77–1.11)	1.42 (1.19–1.69)^***^	1.35(1.10–1.66)^**^	1.45 (1.20–1.76)^***^	0.56 (0.40–0.78)^**^	0.98 (0.76–1.27)	1.04 (0.78–1.39)	1.56 (1.17–2.09)^**^

PR, Prevalence ratio; CI, Confidence interval. All sociodemographic variables were mutually adjusted in multivariate Poisson models. ^*^P < 0.05; ^**^P < 0.01; ^***^P < 0.001.

^a^HK $7.8 = US $1.

^b^Adjusting for sex, age, educational attainment, housing type, monthly household income, and number of cohabitants.

^c^Scale: 0–10, high (7–10), medium (4–6), low (0–3).

## Discussion

This is the first study to examine the prevalence of willingness to attend family services and the associated factors (sociodemographic characteristics, family wellbeing, and family communication quality) among the general population. Such willingness was positively associated with older age, having four or more cohabitants, and higher family wellbeing and communication quality, after adjusting for sociodemographic characteristics. The most popular preferences for family services were healthy living (56.8%), followed by emotion management (47.2%), and family communication promotion (44.1%). We further found that respondents with lower family wellbeing and communication quality preferred activities of emotion management, stress management, family communication promotion, and social network building. This implies that more programs on these topics are needed to promote family relationships.

Lower family wellbeing and family communication quality were negatively associated with the willingness to attend family services. The finding was consistent with the “inverse care law,” which posits that the availability of good medical care tends to vary inversely with the need for it in the population served (12). This is the first study to extend the law to the field of family service—“inverse family care law.” Specifically, respondents who were unwilling to attend family services had lower levels of perceived family harmony, happiness and health. One possible explanation is that respondents with lower family wellbeing and family communication quality were more likely to have physical illness or family conflicts (3, 26), which may dampen their confidence in mending relationships. In China, where family issues are regarded as “best kept inside the house” (27), people may feel shameful to share these unpleasant things with outsiders or seek external professional assistance (28). Promotions for easy-to-access family services are required.

Inconsistent with theories of masculinity revealing men's reluctance to seek help (29), we found that more males than females were willing to attend family services and preferred activities related to social network building. One possible explanation is that men were less likely to provide emotional values in a family (30). Respondents aged 25–44 years showed a high willingness to attend family services, which may partly be due to their inclination to seek professional advice *via* social networking tools (31, 32). Similarly, the higher willingness in respondents with more education could be due to their greater capacity to access supportive resources (31). Inconsistent with the traditional views that low family income is a hindrance to family service use (33), we found respondents with higher monthly household incomes were less willing to attend such services. Future studies are warranted to examine the inconsistent associations between socioeconomic status and the willingness to attend family services.

Over half of the respondents preferred family activities for healthy living, probably due to aging and the COVID-19 pandemic in Hong Kong. Notably, respondents with lower family wellbeing and family communication quality showed higher preferences for social network building. When family stressors (negative events, chronic strains, and trauma) undermine health and wellbeing (7), social support can act as a protective source by cultivating positive interpersonal relationships with others in the family and social community (34, 35).

The “inverse care law” and past literature showed that those with poorer health status are less likely to access care services (12, 13). This is similar to our finding that those with lower family wellbeing and communication quality were less willing to seek professional help from family services. A lower awareness of family or social welfare services was previously shown in the low socioeconomic group (36). Inadequate time to seek professional help was associated with job overburden and homemaking (37). In addition, not knowing or believing that social workers can help may lead to the low use of family services, as the stigma associated with help-seeking (38).

We have first reported an “inverse family care law” regarding the utilization of family services. To promote the utilization of family services in the general population, future interventions could identify those with low levels of family wellbeing and family communication quality using our simple tools and motivate them to seek help as appropriate. Additionally, the most popular preference for family services was related to healthy living (56.8%). These preventive and health promotion activities are usually entertaining and non-stigmatizing, which can help de-stigmatize family service centers. Attracting and engaging more people at risk when they do not have serious problems may motivate them to seek help and remedial services, when they encounter more serious problems in the future.

This study had several limitations. First, causality could not be inferred because of the cross-sectional study design. Second, although we adjusted for several demographic factors, unmeasured confounding factors may have caused biases. For example, people with unpleasant experiences with family services may have a lower willingness to re-attend. Parent-child activities as a family service topic would not be attractive to respondents with no children. Third, this study was conducted during the fourth wave of the COVID-19 pandemic in Hong Kong, during which face-to-face activities were limited to promoting family relationships. Family harmony, happiness, and health worsened in the COVID-19 pandemic (19, 20), thus the prevalence of these have been influenced by the pandemic. Whether the observed associations were affected is uncertain as no pre-pandemic results are available. Finally, in-depth interviews are needed to explore the reasons for the unwillingness to attend family services.

## Conclusions

This study is the first to show the prevalence and associated factors of willingness and preferences to attend family services in a population sample of adults. The findings supported the inverse family care law that people with lower family wellbeing and family communication quality were less willing to attend family services. Future studies are warranted to better understand the unwillingness to attend family services. This could guide the development and promotion of family services and other interventions, particularly for people with low family wellbeing.

## Data availability statement

The datasets presented in this article are not readily available because our analyses and paper writing on the results are in progress. Requests to access the datasets should be directed to the corresponding author.

## Ethics statement

The studies involving human participants were reviewed and approved by Ethics approval was granted by the Institutional Review Board of the University of Hong Kong/Hospital Authority Hong Kong West Cluster (IRB UW 20-651). Informed consent was obtained from all participants included in this study.

## Author contributions

TL, SH, MW, and AL contributed to the study conception and design. WG and SS contributed to the implementation of the program. YZ and WG did the data analysis and wrote the first draft of the manuscript. All authors interpreted the data, participated in the critical review of the report, and provided final approval for publication submission.
