# Supplementary material for: Factors associated with willingness and preferences to attend family services in Hong Kong: A population-based survey
Source: Front Public Health. 2023 Feb 9;11:1057164. doi: 10.3389/fpubh.2023.1057164 (PMC9947657; doi:10.3389/fpubh.2023.1057164)
Supplement: Supplementary file 1 [file Table_1.docx]

**Supplementary Table 1. The associations of willingness to attend family services face-to-face or online with sociodemographic characteristics, family wellbeing and family communication quality, PR (95% CI)**

|  | Services to promote family relationship | | | | Services when facing family problems | | | |
| --- | --- | --- | --- | --- | --- | --- | --- | --- |
|  | Face-to-face, 988/5913 (16.7%) | | Online, 1083/5964 (18.2%) | | Face-to-face, 853/1851 (46.1%) | | Online, 803/1864 (43.1%) | |
|  | Crude model | Adjusted model | Crude model | Adjusted model | Crude model | Adjusted model | Crude model | Adjusted model |
| Sex |  |  |  |  |  |  |  |  |
| Female | 1 | 1 | 1 | 1 | 1 | 1 | 1 | 1 |
| Male | 1.21 (1.08, 1.36) ** | 1.16 (1.03, 1.30) * | 1.21 (1.09, 1.35) ** | 1.16 (1.03, 1.30) * | 1.07 (0.97, 1.18) | 1.07 (0.96, 1.19) | 0.99 (0.90, 1.10) | 0.98 (0.87, 1.09) |
| Age (years) |  |  |  |  |  |  |  |  |
| 18-24 | 1 | 1 | 1 | 1 | 1 | 1 | 1 | 1 |
| 25-44 | 2.07 (1.52, 2.81) *** | 2.45 (1.72, 3.47) *** | 2.20 (1.65, 2.93) *** | 2.55 (1.84, 3.54) *** | 1.64 (1.25, 2.15) *** | 1.69 (1.24, 2.31) ** | 1.46 (1.13, 1.90) ** | 1.52 (1.13, 2.04) ** |
| 45-64 | 2.08 (1.53, 2.82) *** | 2.40 (1.68, 3.43) *** | 2.05 (1.54, 2.74) *** | 2.45 (1.75, 3.42) *** | 1.65 (1.26, 2.16) *** | 1.66 (1.21, 2.27) ** | 1.48 (1.14, 1.92) ** | 1.52 (1.13, 2.05) ** |
| ≥65 | 2.28 (1.65, 3.16) *** | 2.39 (1.62, 3.52) *** | 1.57 (1.14, 2.17) ** | 1.91 (1.31, 2.79) *** | 1.53 (1.14, 2.05) ** | 1.57 (1.11, 2.23) * | 1.09 (0.81, 1.48) | 1.26 (0.89, 1.77) |
| Educational attainment |  |  |  |  |  |  |  |  |
| Secondary or below | 1 | 1 | 1 | 1 | 1 | 1 | 1 | 1 |
| Tertiary | 0.97 (0.85, 1.10) | 1.08 (0.92, 1.25) | 1.30 (1.14, 1.49) *** | 1.30 (1.11, 1.52) ** | 1.00 (0.90, 1.12) | 0.95 (0.83, 1.10) | 1.10 (0.97, 1.24) | 1.01 (0.87, 1.16) |
| Housing type |  |  |  |  |  |  |  |  |
| Rented | 1 | 1 | 1 | 1 | 1 | 1 | 1 | 1 |
| Owned | 0.99 (0.88, 1.12) | 0.99 (0.87, 1.12) | 1.02 (0.91, 1.14) | 0.99 (0.88, 1.12) | 0.98 (0.89, 1.08) | 0.95 (0.85, 1.06) | 1.05 (0.94, 1.17) | 1.00 (0.89, 1.12) |
| Monthly household income (HK$) ^a^ | |  |  |  |  |  |  |  |
| ≤9,999 | 1 | 1 | 1 | 1 | 1 | 1 | 1 | 1 |
| 10,000-39,999 | 0.75 (0.62, 0.90) ** | 0.72 (0.59, 0.88) ** | 0.94 (0.78, 1.14) | 0.81 (0.66, 1.00) * | 1.08 (0.89, 1.31) | 1.02 (0.84, 1.25) | 1.18 (0.96, 1.45) | 1.03 (0.83, 1.27) |
| ≥40,000 | 0.81 (0.68, 0.96) * | 0.73 (0.60, 0.89) ** | 1.03 (0.86, 1.23) | 0.78 (0.64, 0.96) * | 1.23 (1.02, 1.47) * | 1.13 (0.93, 1.39) | 1.32 (1.08, 1.60) ** | 1.10 (0.89, 1.36) |
| Number of cohabitants |  |  |  |  |  |  |  |  |
| 0 | 1 | 1 | 1 | 1 | 1 | 1 | 1 | 1 |
| 1-3 | 1.32 (1.04, 1.67) * | 1.19 (0.93, 1.53) | 1.24 (1.00, 1.54) | 1.15 (0.90, 1.46) | 1.34 (1.08, 1.66) ** | 1.38 (1.06, 1.80) * | 1.65 (1.27, 2.15) *** | 1.73 (1.25, 2.40) ** |
| ≥4 | 1.52 (1.17, 1.99) ** | 1.44 (1.08, 1.91) * | 1.45 (1.14, 1.86) ** | 1.38 (1.05, 1.82) * | 1.33 (1.04, 1.71) * | 1.35 (1.00, 1.82) * | 1.69 (1.26, 2.26) *** | 1.78 (1.26, 2.53) ** |
| Family wellbeing ^b, c^ |  |  |  |  |  |  |  |  |
| High | 1 | 1 | 1 | 1 | 1 | 1 | 1 | 1 |
| Medium | 0.81 (0.71, 0.93) ** | 0.82 (0.71, 0.95) ** | 0.85 (0.75, 0.97) * | 0.84 (0.73, 0.97) * | 0.78 (0.69, 0.87) *** | 0.81 (0.71, 0.91) ** | 0.80 (0.71, 0.90) *** | 0.82 (0.72, 0.93) ** |
| Low | 0.73 (0.56, 0.95) * | 0.71 (0.53, 0.95) * | 0.69 (0.53, 0.89) ** | 0.64 (0.48, 0.85) ** | 0.45 (0.32, 0.62) *** | 0.44 (0.31, 0.63) *** | 0.43 (0.31, 0.61) *** | 0.44 (0.30, 0.64) *** |
| Family communication quality ^b, c^ | | | | | | | | |
| High | 1 | 1 | 1 | 1 | 1 | 1 | 1 | 1 |
| Medium | 0.88 (0.78, 0.99) * | 0.90 (0.79, 1.02) | 0.90 (0.80, 1.01) | 0.89 (0.79, 1.01) | 0.83 (0.75, 0.92) *** | 0.82 (0.74, 0.92) ** | 0.85 (0.76, 0.95) ** | 0.87 (0.77, 0.98) * |
| Low | 0.64 (0.52, 0.78) *** | 0.65 (0.52, 0.81) *** | 0.74 (0.62, 0.89) ** | 0.71 (0.58, 0.87) ** | 0.47 (0.38, 0.58) *** | 0.45 (0.36, 0.58) *** | 0.60 (0.49, 0.73) *** | 0.59 (0.48, 0.74) *** |

Note: PR=prevalence ratio, CI=confidence interval. All sociodemographic variables were mutually adjusted in multivariate Poisson models. ^*^ P<0.05; ^**^ P<0.01; ^***^ P<0.001

^a^ HK $7.8 = US $1

^b^ Adjusting for sex, age, educational attainment, housing type, household income and number of cohabitants.

^c^ Scale: 0-10, high (7-10), medium (4-6), low (0-3).
